# 2021年毛细管电泳技术年度回顾

**DOI:** 10.3724/SP.J.1123.2022.03040

**Published:** 2022-07-08

**Authors:** Yao MA, Yangyang HU, Liting ZHENG, Li CHEN, Xinying ZHAO, Feng QU

**Affiliations:** 1.北京理工大学生命学院, 北京 100081; 1. School of Life Science, Beijing Institute of Technology, Beijing 100081, China; 2.北京电子科技职业学院, 北京 100176; 2. Beijing Polytechnic, Beijing 100176, China

**Keywords:** 毛细管电泳, 2021, 年度回顾, capillary electrophoresis (CE), 2021, annual review

## Abstract

该文为2021年毛细管电泳(capillary electrophoresis, CE)技术年度回顾。归纳总结了以“capillary electrophoresis-mass spectrometry”或“capillary isoelectric focusing”或“micellar electrokinetic chromatography”或“capillary electrophoresis”为关键词在ISI Web of Science数据库中进行主题检索(排除“capillary electrochromatography”“microchip”和“capillary monolithic column”)得到的2021年CE技术相关研究论文291篇,以及中文期刊《色谱》和《分析化学》中相关研究论文9篇。重点介绍了影响因子(IF)≥10.0的*Coordination Chemistry Reviews*, *Angewandte Chemie-International Edition*, *Nature Protocols*, *TrAC-Trends in Analytical Chemistry*, *Signal Transduction and Targeted Therapy*发表的7篇论文;以及影响因子5~10之间的代表性期刊*Analytical Chemistry*, *Analytica Chimica Acta*, *Talanta*和*Food Chemistry*的42篇论文;对影响因子小于5但CE技术报道较为集中的*Journal of Chromatography A*和*Electrophoresis*,国内重要的中文期刊《色谱》和《分析化学》中的代表性工作进行了概述。该文根据国际通用学术水平评价指标之一的影响因子选择期刊,结合期刊发表CE论文代表性工作进行介绍,便于读者快速了解毛细管电泳技术在过去一年的重要研究进展。

## Abstract (en)

This paper provides an annual review of capillary electrophoresis (CE) technology in 2021. A total of 291 research papers related to CE technology published in 2021 were retrieved from the ISI Web of Science using the keywords, “capillary electrophoresis-mass spectrometry” “capillary isoelectric focusing” “micellar electrokinetic chromatography”, or “capillary electrophoresis” (not “capillary electrochromatography” “microchip” and “capillary monolithic column”). In addition, nine research papers related to CE technology in Chinese journals were reviewed: *Chinese Journal of Chromatography* and *Chinese Journal of Analytical Chemistry*. This review focused on seven papers published in *Coordination Chemistry Reviews*, *Angewandte Chemie-International Edition*, *Nature Protocols*, *TrAC-Trends in Analytical Chemistry*, and *Signal Transduction and Targeted Therapy* with impact factors (IFs) greater than 10.0, as well as 42 papers reported in *Analytical Chemistry*, *Analytica Chimica Acta*, *Talanta*, and *Food Chemistry* with IFs between 5.0 and 10.0. This review also provides a comprehensive overview of representative CE works in *Journal of Chromatography A* and *Electrophoresis* with IFs<5.0, as well as important Chinese journals, *Chinese Journal of Chromatography* and *Chinese Journal of Analytical Chemistry*. According to the IF, this paper introduces the representative work of CE-related papers to allow readers to quickly understand the important research progress of CE technology in the past year.

截至2021年12月31日,以“capillary electrophoresis-mass spectrometry”或“capillary isoelectric focusing”或“micellar electrokinetic chromatography”或“capillary electrophoresis”为关键词在ISI Web of Science数据库中进行主题检索(排除“capillary electrochromatography”“microchip”和“capillary monolithic column”),检索到期刊论文共计291篇。

IF≥10.0的部分顶级期刊*Coordination Chemistry Reviews*(IF=22.3), *Angewandte Chemie-International Edition*(IF=15.3), *Nature Protocols*(IF=13.5), *TrAC-Trends in Analytical Chemistry*(IF=12.3)和*Signal Transduction and Targeted Therapy*(IF=12.9)中发表了7篇“毛细管电泳(CE)”相关论文。10.0>IF≥5.0的24种期刊中发表了CE相关论文108篇,*Analytical Chemistry*(IF=6.9)发表20篇,*Analytica Chimica Acta*(IF=6.5)发表16篇,*Talanta*(IF=6.0)发表25篇,影响力较大的食品分析期刊*Food Chemistry*(IF=7.5)发表6篇。IF<5.0的16种期刊中发表了CE相关论文176篇,与CE关联密切的*Journal of Chromatography A*(IF=4.7)和*Electrophoresis*(IF=3.5)分别发表41篇和47篇。中文重点期刊《色谱》8篇、《分析化学》1篇。

本文根据国际通用学术水平评价指标之一IF选择期刊,结合期刊发表CE论文代表性工作进行介绍,便于读者快速了解毛细管电泳技术在当年的重要研究进展。

## 1 IF≥10.0期刊

### 1.1 新方法和新应用

Ryšavá等^[1]^在*Angewandte Chemie-International Edition*发表了毛细管电泳-紫外检测法(CE-UV)结合干血点(dried blood spot, DBS)定量分析血液样本中的抗凝血药华法林的方法。取指血,DBS处理,封装,直接快递至实验室;处理至可以上机的过程共需680 s(其中80 μL乙腈提取180 s,其他为辅助处理)。CE分离条件为30 mmol/L醋酸+30 mmol/L醋酸钠+30% (v/v)乙腈水溶液,pH 5.2,检测波长205 nm, 520 s后完成定量测定。该法用于临床血液快速分析,样品收集方便快捷,定量全时长20 min,展示了优秀的分析性能,是一种监测人体健康的有力工具。Shanmuganathan等^[2]^在*Nature Protocols*发表了基于多段注射-毛细管电泳-质谱法(MSI-CE-MS)建立的大规模流行病学血清代谢组学研究的高通量平台和标准化数据流程。通过连续注射后进行多重分离,一次可分析7个独立样品。多元复合分离提高了血清中66种极性离子代谢物的定量测定通量(< 4 min/样品)、变异系数(< 30%)、高频率(>75%)(*n*=1004)。代谢产物与临床分析具有一致性(平均偏差=11%, *n*=668),回收标准(平均变异系数=12%, *n*=2412)。此外,还报告了53种不同种族孕中期血清代谢物的参考间隔。Yang等^[3]^在*Signal Transduction and Targeted Therapy*发表了血清基质辅助CE结合指数富集的配体系统进化技术(CE-SELEX),通过3轮筛选获得新冠病毒S1蛋白的适配体,该适配体对S1蛋白具有抑制作用,并可用于检测新冠病毒。

### 1.2 重要综述

*TrAC-Trends in Analytical Chemistry*发表的3篇论文介绍了CE技术在病原体、医用蛋白质和细菌分析的巨大潜力。Buszewski等^[4]^基于电迁移技术结合蛋白质组、脂质组和基因组的激光解吸/电离分析同步分析,提出了病原体预分离的新方法,实现微生物可分离组分的快速筛选和低成本分组,并将其作为一种快速检测和鉴定的方法,用于SARS-COV-2的临床监测、疾病预防和治疗决策。Kaur等^[5]^聚焦治疗性蛋白质分析和表征,综述了常规释放试验、稳定性试验和深度表征方法的最新应用进展,对十二烷基硫酸钠毛细管电泳法(SDS-CE)、毛细管区带电泳(CZE)和图像化毛细管等电聚焦(iCIEF)等用于单克隆抗体和相关治疗药物的大小异质性、糖基化异质性和电荷异质性表征方法的优缺点进行了重点讨论。Kartsova等^[6]^综述了电动法与各种检测器及在线浓缩技术结合,用于细菌代谢物、细胞成分和细菌分析的最新进展,讨论了获得更高分辨率电泳系统的改进方法和细菌作为背景电解质和毛细管壁改进剂的应用,提供了细菌样本测试方法的完整应用程序和实用性建议。此外,Zhang等^[7]^在*Coordination Chemistry Reviews*上综述了近年来应用于CE的各种手性功能材料的研究进展和发展现状,介绍了离子液体(ILs)、深共晶溶剂(DESs)、纳米粒子(NPs)、分子印迹聚合物(MIPs)、金属有机骨架(MOFs)、共价有机骨架(COFs)等最具代表性的先进功能材料,在优化CE的核心要素缓冲溶液条件(背景电解质的种类和浓度、pH、溶剂、手性选择剂等)、分离模式(胶束电动毛细管色谱(MEKC)、非水毛细管电泳技术(NACE)等)和仪器参数(毛细管内径和长度、温度、速度、进样模式、检测器)三方面的应用。将功能性材料引入手性物质分析是解决传统手性分析难题的新方法。

此外,近十年,部分顶级期刊发表的论文具有一定的导向性和实用性(见表1),打消了读者对毛细管电泳技术不能有好应用、不能做出好方法、不能发表好文章的顾虑,为毛细管电泳技术在其他行业和领域的应用提供了有价值的参考。

**表 1:** 部分顶级期刊发表毛细管电泳技术相关论文数量

Journal	2012	2013	2014	2015	2016	2017	2018	2019	2020	2021
Nature Communications						1		1	1	
Nature Chemical Biology					1					
Nature Protocols		1				1				1
Chemical Science				1		1		1	1	
Small									1	
Coordination Chemistry Reviews										1
Angewandte Chemie-International Edition	1		3				1	1		1
Signal Transduction and Targeted Therapy										1
TrAC-Trends in Analytical Chemistry	3	3	3	1		1	3	3	1	3
Biotechnology Advances								1		
PLoS Medicine									1	

## 2 10.0>IF≥5.0期刊

### 2.1 *Analytical Chemistry*

#### 2.1.1 CE的新方法和新应用

新材料和毛细管凝胶电泳技术(CGE) Crihfield等^[8]^制备了由普通磷脂自组装而成的热响应纳米凝胶,表观孔径为20%、25%和30%的纳米凝胶可用于分离相对分子质量为20.5~79.5 kDa的蛋白质,半径范围为1.8~4.8 nm,这种更为精确的尺寸阶梯将解决与某些蛋白质的非球面几何形状有关的问题,大大提高量化的精确性。此外,Brooijmans等^[9]^开发了两种基于NACE的方法:脱质子(用有机可溶的强碱使羧酸官能团脱质子化)和异位共轭(用羧酸官能团与羧酸阴离子进行杂共轭)来分离酸功能聚合物。研究表明脱质子化方法有效聚合物迁移率大约是异位共轭方法的2倍,后者的稳定性优于前者。Bwanali等^[10]^通过毛细管纳米凝胶电泳方法测定了*β*-1,4半乳糖基转移酶Michaelis-Menten常数,可实时转移半乳糖残基到*N*-聚糖和糖蛋白,且已通过赫赛汀(Herceptin)糖蛋白成功验证,经酶修饰后的刺桐凝集素完全阻滞证实了半乳糖残基在Herceptin糖蛋白中的添加。此外,Filep等^[11]^利用SDS-CGE研究单体(右旋糖酐)和交联剂(硼酸盐)比例对单克隆抗体亚基分离选择性的影响。研究结果表明,较低的单体/交联剂比例凝胶组分可以更好地分离糖基化和非糖基化的单克隆抗体重链片段,高浓度行业标准凝胶配方对非糖基化物具有更好的分离效果。Guttman等^[12]^探索了不同质量分数的右旋糖酐单体(2%、5%、7.5%和10%)和硼酸酯交联剂(2%、4%)对SDS-CGE分离蛋白质标准混合物的影响,广泛使用的10%右旋糖酐和4%硼酸盐凝胶是最佳的选择,可以在必要时为特定的分离进行配方优化。

新探针和亲和电泳技术(ACE) Li等^[13]^通过使用三重功能DNA荧光探针检测地高辛和赭曲霉毒素A,通过使用两个不同长度和不同流动性的DNA探针,在一次CE运行中同时检测2个目标,实现了抗体结合、荧光记录和分离,检出限水平为nmol/L。Davoine等^[14]^通过间接亲和毛细管电泳(iACE)表征了凝血因子XIIa片段结合物、解离常数*K*_d_以及片段结合位点,提出了新方法使结合位点图形化,进一步加深了iACE在药物化学领域的探索深度。

Zhu等^[15]^利用CE技术筛选蛋白质的适配体,研究了核酸库随机区域长度与适配体亲和力的相关性,针对不同蛋白质,随机区域长度是影响适配体亲和力的关键因素之一,筛选时也应注意聚合酶链式反应(PCR)过程对筛选效率的影响,首次报道了压力感应蛋白piezo2的核酸适配体。

#### 2.1.2 CE-MS的新硬件和新应用

硬件研发 Huang等^[16]^开发了电喷雾辅助装置喷雾毛细管(<15 μm的锥形毛细管),消除了离线样品处理步骤带来的潜在污染和样品损失,且稳定性和重现性强,pL~nL级进样,可通过更小的激光拉尖(8 μm或更小)设计装置用于不同类型的单细胞样本(如人类细胞系),虽然目前依赖于手动操作,但在单细胞代谢组学领域依旧具有巨大潜力。Schlecht等^[17]^开发了基于3D打印技术的新型纳米流鞘液体毛细管电泳-质谱(nanoflow SL CE-MS)接口,即插即用,灵活性强(使用3D打印可以轻松调整和优化打印部件),用于打印的材料化学稳定性强、透明度高,便于毛细管插入和故障排除,且双毛细管方法的实施便于频繁地重涂静态吸附涂层以防样品吸附,最大限度地加强了迁移时间稳定性。Zamuruyev等^[18]^开发了全自动便携式毛细管电泳系统,微流体和气动回路与旋转阀相结合的原始设计解决了毛细管电泳仪器最具挑战性的问题:流体回路各部分之间的高压隔离、精确的样品进样以及所有步骤的自动化;与电喷雾质谱法耦合的自动化CE系统为未来的航天应用奠定基础。

用于单细胞水平的表征 Jooβ等^[19]^开发了原生毛细管区域电泳-自上而下质谱法(nCZE-TDMS),进行了10^-18^单核细胞小体(Nucs)表征。在线分离Nucs后,采用三重四极杆质谱方法,测定Nucs的完整质量,喷射并检测组蛋白,并对组蛋白片段进行排序。推动了染色质和表观遗传学领域核小体水平的研究,有潜力成为表观遗传学、天然蛋白质组学以及复杂合成生物分子的质量控制领域的重要工具。Mast等^[20]^开发了毛细管电泳-热离子质谱技术(CE-TIMS),在单细胞水平上表征肽和肽二聚体,测定了从加利福尼亚海兔中枢神经系统分离的单个神经元中提取的3个神经肽基因产物的立体化学构型,使单细胞和神经组织中肽非对映体的立体化学表征和相对定量成为可能。Han等^[21]^开发了自动化的免疫亲和-毛细管电泳-质谱(IA-CE-MS)全面综合平台,用于表征广泛的生物治疗药物,包括多肽、单克隆抗体和双特异性抗体,在药物发现的早期阶段进行生物转化研究,可以加快药物开发过程。

用于组学分析 Chen等^[22]^首次将CZE-MS/MS技术用于大规模的自上而下组蛋白组学(组蛋白TDP),从不到300 ng的蛋白质材料中鉴定出近400种组蛋白变体,与反相液相色谱-串联质谱(RPLC-MS/MS)相比,CZE-MS/MS灵敏度更高,是一种在蛋白质组学水平上描述组蛋白编码的有效替代方法;但是已鉴定的组蛋白变体的裂解覆盖率有限,可通过基于电子或光子的裂解方法(如电子捕获解离(ECD)、电子转移解离(ETD)或紫外光解离(UVPD))以及CZE-MS/MS技术来提高裂解覆盖率。Gstöttner等^[23]^通过ACE-MS技术表征样本中单个抗体蛋白质组与新生儿Fc受体(FcRn)的亲和力,研究了FcRn与抗体的相互作用和结合化学计量比,证明了具有不同结合亲和力(即氧化型)的蛋白质在FcRn存在下表现出不同的电泳迁移率。Marie等^[24]^优化了CZE-ESI-MS技术的*N*-聚糖分析方法,用于微量来源于人类血液的免疫球蛋白G(IgG)和血浆总胞外囊泡(EV)分离物释放的*N*-聚糖的高灵敏度表征,大大加强了表征深度(鉴定的*N*-聚糖数量增加了5倍)。Delvaux等^[25]^利用CE-MS技术对细菌细胞质提取物中3个肽聚糖(PNG)及其酰胺化衍生物进行定量分析,结果重复性好,相对标准偏差(RSD)接近1%,包括生物处理在内的方法整体重现性优于20%。

#### 2.1.3 综述性论文

Tobolkina等^[26]^概述了CE在发展中国家药物质量控制中的应用优势(便携、绿色经济、检出限低)和尚未解决的各种问题;指出未来应该改进方法技术,建立高效管理和信息共享的数据库和开发在线教育课程;呼吁使用者支持更变便捷方式,如智能手机拍照、对产品进行地理定位以及创建和管理数据库来收集药物信息。Wang等^[27]^概述了2018~2020年亲和毛细管电泳技术(ACE)的代表性论文,对基本原理、亲和力相互作用、热力学和动力学参数、方法开发以及新应用做了综述。指出迁移率移位亲和毛细管电泳(mobility shift ACE)和预平衡亲和毛细管电泳(pre-equilibrium ACE)在测量一系列结合强度的相互作用方面非常具有应用前景,动态毛细管电泳(kinetic capillary electrophoresis, KCE)可扩大ACE的应用范围,而部分填充亲和毛细管电泳(partial-filling ACE)则适用于低样本消耗的亲和力相互作用测量。

### 2.2 *Analytica Chimica Acta*

#### 2.2.1 CE的电渗流(EOF)调节

Konášová等^[28]^通过共价阳离子共聚涂层调节电渗流进而优化毛细管电泳分离过程,用CE-UV和纳米喷雾CE-MS对乙酸基酸性背景电解质(BGE)中系列基础药物分子的反电渗流现象进行表征分析,证明可调EOF的共价阳离子共聚涂层是优化分辨率、分离效率和分析物迁移时间的有效工具。Nguyen等^[29]^开发了通过调节EOF来实现更好的毛细管电泳电动预富集的新方法,无需涂层,将缓冲液浓度增加到非常高的水平(超过1000 mmol/L)即可。用于核壳磁性纳米粒子(CSMNPs)的测定,灵敏度提高了近350倍,该方法在医疗保健应用方面具有很高的潜力。

#### 2.2.2 CE-MS用于生物样本中的纳米颗粒研究

Labied等^[30]^采用泰勒色散分析-电感耦合等离子体质谱(TDA-ICP-MS)和CE-ICP-MS相结合的方法研究临床2期超微含钆纳米颗粒AGuIX的降解途径。测量得到的纳米颗粒尺寸和在不同介质(磷酸盐缓冲液、尿液和血清)中的钆形态表明,AGuIX在血清中的溶解速度加快,在每种介质中都没有释放游离钆。Men等^[31]^将CE-ICP-MS结合高性能螺旋流喷雾室(SFSC)的模式用于溶解Ag(I)和银纳米颗粒(AgNPs)胞内形态的研究,LOD为87 ng/L,相对峰面积的RSD<3%,迁移时间的RSD<2%,细胞裂解液中峰值回收率为92.7%~106.6%。由于癌细胞内的氧化应激或酸性微环境,AgNPs在细胞内部的溶解度高于在培养基中的溶解度。

#### 2.2.3 综述性论文

Liénard等^[32]^综述了近15年不同模式的电泳技术用于样品处理、分离和定量的液滴界面策略,重点介绍了不互溶相液滴、基于介电原理的电润湿数字微流体和喷墨液滴生成。Ta等^[33]^综述了20年间CE用于氨基酸分析的研究进展,毛细管电泳激光诱导荧光法(CE-LIF)的检出限水平为nmol/L,毛细管电泳电导法(CE-CD)可应用于大多数氨基酸的检测,通过CE技术很容易实现氨基酸对映体的分离。值得关注的是,Yang等^[34]^首次将电容耦合非接触电导检测(C^4^D)和光度检测(PD)相结合并使用3D打印技术制备了二合一毛细管检测器,PD检测显示出良好的线性关系,杂散光下降到8%,有效光路长度为73%,通过CE对4-(2-吡啶偶氮)间苯二酚络合物中阳离子的分离和检测,验证了该方法的可行性。

### 2.3 *Talanta*

#### 2.3.1 CE用于快速分析

Wu等^[35]^利用基于非固定化细胞毛细管电泳(NICCE)的方法,结合数学模型对单个肿瘤细胞的叶酸受体(FRs)进行量化,通过NICCE分别研究了叶酸(FA)与A549、HT-29、HepG2和U87MG细胞的相互作用,计算了动力学参数吸附平衡常数(*K*_a_)、保留因子(*k*)、结合速率常数(*k*_a_)和*K*_d_,建立了数学模型,精确地确定FRs的浓度,结果与酶联免疫吸附(ELISA)试验的结果一致,简单、快速、灵敏,且无需分离纯化蛋白质,大大加快了分析速度,有望实现单细胞细胞膜受体表达水平的临床检测。Vieira等^[36]^介绍了CZE中多次进样进行内部标准化的新技术——外塞标准化(OPS),将该技术应用于雨水样品中氯化物、硝酸盐和硫酸盐的测定,分离时间小于1 min,氯化物、硝酸盐和硫酸盐的LOD分别为0.05、0.09和0.11 mg/L,对82个雨水样本的结果进行了统计,95%置信水平内未发现统计差异。Zhu等^[37]^首次报道了甲状腺球蛋白(Tg)的核酸适配体,*K*_d_为4.51 nmol/L,可用于纳米金比色分析。

#### 2.3.2 CE-MS增敏技术

Konášová等^[38]^利用石英发射器蚀刻处理过的熔融石英毛细管,构建纳米流鞘液CE-ESI-MS界面,通过螺纹交叉耦合用于鞘液和电极连接,测试界面在分离毛细管和ESI发射管尖端的不同位置将ESI电势与分离电势解耦的能力,为所选药物和苄基吡啶阳离子的CE-ESI-MS分析提供了更高的灵敏度。Pérez-Alcaraz等^[39]^首次提出固相萃取与毛细管电泳-质谱法(SPE-CE-MS)在线联用技术,用于测定尿液中*R*,*S*-3,4-亚甲基二氧吡戊酮(*R*,*S*-MDPV)的对映体,以含有0.5%硫酸化-*α*-CD的10 mmol/L醋酸铵BGE(pH 7)为手性选择剂,在线性范围30~250 ng/mL内,LOD为10 ng/mL。Irfan等^[40]^利用氧化钛涂层核壳介孔二氧化硅(CSMS@TiO_2_)纳米复合材料(直径1.0 μm、壳厚120 nm、比表面积77 m^2^/g、孔容0.15 cm^3^/g)提高CE-MS鉴定磷酸肽的灵敏度,用于分析*β*-酪蛋白胰蛋白酶消化物和牛血清白蛋白(BSA)蛋白质混合物,检测到的磷酸肽数量显著增加,即使在极低浓度的*β*-酪蛋白(1 fmol/μL)下也可以实现磷酸肽的富集。

#### 2.3.3 综述性论文

Acquavia等^[41]^综述了生物体液和组织中COVID-19抗病毒药物样品的预处理和提取方法,检测和定量方法,讨论了这些方法的当前趋势、优缺点和前景,为建立最合适的检测方法以帮助对抗COVID-19病毒造成的危害提供有价值的参考。Lyu等^[42]^综述了捕获指数富集的配体系统进化技术(capture-SELEX)的最新研究进展,针对无法固定的靶点(尤其是小分子靶点)筛选适配体,虽然可能存在误报和缺乏多样性,但仍具有巨大的潜力。Seyfinejad等^[43]^总结了测定游离药物浓度及其与血浆蛋白结合的分析方法的最新进展,发现提供高通量分析的表面等离子共振法(SPR)生物传感器适用于大药物分子,高效亲和色谱法(HPAC)和毛细管电泳-前沿分析(CE-FA)适用于极性不同的小型和大型药物尤其是低亲水性(疏水性)分子,预测具有高特异性和配备灵敏探测器的在线方法将成为该方面研究的大热趋势。

### 2.4 *Food Chemistry*

#### 2.4.1 CE的应用

Galindo-Luján等^[44]^通过毛细管电泳-紫外检测-二极管阵列检测(CE-UV-DAD)对藜麦中的蛋白质指纹识别分类,获得不同藜麦品种的可溶性蛋白质提取物的特征多波长电泳图谱,应用多变量曲线分辨率交替最小二乘法(MCR-ALS)、主成分分析(PCA)和偏最小二乘判别分析(PLS-DA)对电泳图进行反卷积,用于含藜麦食品的质量控制。Aredes等^[45]^通过CZE直接测定了麦汁和啤酒样品的氨基酸含量,监测了啤酒的糖化阶段,分析了发酵的最终产物,结果均符合预期,表明CZE与PCA结合可有效评估精酿啤酒质量。Seraglio等^[46]^通过CE获得了不同年份花蜜和糙叶含羞草蜜露蜂蜜(BHH)中脂肪族有机酸(AOA)的重要数据,得出葡萄糖酸是这两种蜂蜜中的主要AOA, BHH通常比花蜜含有更高含量的AOA,与收获年份无关;可用于区分花蜜,以及纯的和掺假的BHH,有助于识别可能与花蜜混合的BHH。此外,Sarkozy等^[47]^利用12通道毛细管凝胶电泳技术以常规的碳水化合物分离基质和硼酸盐交联的葡聚糖凝胶,可同时分析12个样品,20 min完成人乳寡糖(HMO)的分离和分析。

#### 2.4.2 CE-MS的应用

Moreno-González等^[48]^通过在线分子印迹聚合物固相萃取-毛细管电泳耦合串联质谱法(MISPE-CZE-MS/MS)测定苹果样品中的棒曲霉素,LOQ为1 μg/kg,相关系数*R*^2^ ≥0.997,灵敏度、通量和自动化程度更高,且在测定苹果饮料中棒曲霉素时不受主要干扰物5-羟甲基糠醛(5-HMF)干扰,具有应用于苹果产品常规分析的潜力。Perez等^[49]^首次利用CE-MS/MS测定炊具中19种初级芳香胺(PAA),运行时间低于6 min, *R*^2^≥0.99、LOD为0.2~1.3 μg/kg,回收率为85%~120%,可以用于炊具中PAA含量的测定。

## 3 IF<5.0的重要期刊

### 3.1 *Journal of Chromatography A*

#### 3.1.1 CE用于药物研发和质控

Song等^[50]^建立了CZE在线分离、定量口蹄疫病毒(FMDV)单价疫苗和二价疫苗抗原的方法,再现性RSD < 5%, *R*^2^=0.999。方法定量分析FMDV的各血清型,不受核酸杂质干扰,具有进一步推广到其他多价病毒和病毒疫苗质量控制的潜力。Dadouch等^[51]^首次在分离毛细管中进行了多次在线反应(同时还原和消化),用于中上层单克隆抗体质量控制,证实了CZE在整合样品制备和多个分离步骤方面的实用性,以及不同条件下分析的高度灵活性,所开发的条件符合质谱分析要求,为无鞘CZE-MS进行完整的单克隆抗体表征奠定了基础。Michalcová等^[52]^利用CE-FA技术在线测定血浆蛋白-药物结合参数,测定了模型药物普萘洛尔(PRO)、利多卡因(LIDO)和苯丁氮酮(PHE)的BSA表观结合参数,与文献^[53⇓⇓-56]^数据一致,具有较大的潜在价值。Lou等^[57]^利用非平衡毛细管电泳(NECEEM)筛选抑制泛素样含PHD和环指域蛋白1(UHRF1)-甲基化DNA(mDNA)复合物形成的抑制剂,可在一次实验中测量所有的平衡和动力学参数,同时发现了天然的原花青素和黄芩素可以作为UHRF1-mDNA的新抑制剂。

#### 3.1.2 CE-MS用于临床样本研究

Piestansky等^[58]^利用二维毛细管异速电泳-CE-MS超灵敏定量真实人尿样品中的血清素,检出限为34 pg/mL, *R*^2^> 0.99,日内精密度和日间精密度RSD为3.5%~12.2%,相对误差为99%~109.4%,回收率为96%~102%,可成为常规临床筛查或尿液样本中血清素的目标代谢组学研究的有用工具。Igarashi等^[59]^利用多段注射毛细管电泳-三重四极杆质谱(MSI-CE-MS/MS)高通量筛选唾液中的多胺,可区分结直肠癌患者和非癌症对照者;40 min内可分析唾液标本40份,速度快,选择性强,灵敏度高,具有良好的重复性、线性和定量准确度,有望在实验室检测中应用于直肠癌的筛查。Nam等^[60]^比较了CE-MS/MS和GC-MS/MS分析血清的磷酸化化合物,二者测定的甘油3-磷酸(G3P)在细胞提取物中的浓度表现出一定的差异,但前者的分析范围更广(加标血清样品中所有15种磷酸化化合物),检出限0.25~2 μmol/L高于后者0.05~0.5 μmol/L。

### 3.2 *Electrophoresis*

#### 3.2.1 手性CE和CE-C^4^D

Urlaub等^[61]^通过手性毛细管电泳法研究了右旋布洛芬在球磨机中的异构化,结果显示只有在特定条件下才能观察到异构化,而在片剂、胶囊和软膏中不会发生异构化,因此药物制剂在储存时很可能是稳定的。Sandbaumhüter等^[62]^收集设得兰矮种马注射美沙酮药物前后的动脉血样品,使用高度硫酸化的*γ*-环糊精作为手性选择剂,通过手性毛细管电泳法研究了外消旋美沙酮代谢后的立体异构的变化。结果显示,D-美沙酮和D-2-亚乙基-1,5-二甲基-3,3-二苯基吡咯烷(D-EDDP)的清除速度比它们各自的L-对映体快,为监测少量血浆中美沙酮及其代谢产物的对映异构体提供了有力的工具。Mikkonen等^[63]^首次用CE-C^4^D分析哺乳动物细胞培养上清液中的氨基酸,可以监测细胞培养过程中17种氨基酸的浓度变化,分析时间、样品和试剂消耗都显著降低。T $\overset{{}^{\circ}}{u}$ ma等^[64]^通过CE-C^4^D技术监测胰腺导管腺癌(PDAC)和癌症恶病质(CC)患者的循环氨基酸(支链氨基酸(BCAA)、丙氨酸和谷氨酰胺),结果表明,患有PDAC和CC的患者基础循环BCAAs和谷氨酰胺水平低,胰岛素依赖性循环水平抑制减弱,这是CC合成代谢阻力的一个特征,值得深入研究。

#### 3.2.2 CE-MS与其他技术联用

Wang等^[65]^通过流动的微孔界面将CE与MS联用以提高ESI的稳定性和检测灵敏度,测量了9种氨基酸和5种肽的关键性能指标,与成熟的商用鞘流界面相比,具有较大的优势。Wang等^[66]^将ESI-MS、CE-FA和TDA联用,研究人类癌基因c-KIT启动子G4和天然黏合剂之间的相互作用,与传统的结合方法相比,分析速度、准确性和特异性均有提升,有望在快速寻找新一代G4靶向黏合剂中发挥重要作用。Kumar等^[67]^利用LC-CE-MS/MS分析产生单克隆抗体的中国仓鼠卵巢(CHO)细胞系中的宿主细胞蛋白质(HCPs),可在更大的尺寸、等电点(pI)和疏水性范围内鉴定肽,相对于传统一维方法可识别更多HCPs,该法将成为HCPs分析和产生单克隆抗体的CHO细胞分泌组研究的有用补充。

十年来,CE技术在应用方面更为广泛和成熟(见表2)。

**表 2:** 重要期刊发表毛细管电泳技术相关论文数量

Journal	2012	2013	2014	2015	2016	2017	2018	2019	2020	2021
Analytical Chemistry	16	17	11	24	22	28	15	14	9	20
Analytica Chimica Acta	10	47	15	12	21	12	12	15	5	16
Talanta	41	33	42	13	21	19	22	21	10	25
Food Chemistry	8	4	8	12	7	9	2	3	5	6
Journal of Chromatography A	30	89	54	39	40	42	42	23	16	41
Electrophoresis	77	81	71	65	99	72	57	63	24	47
Total	398	362	379	427	458	450	354	348	222	291

## 4 国内进展

### 4.1 《色谱》

王源豫等^[68]^制备了基于智能手机的便携式毛细管电泳装置,集成了C^4^D和蓝牙通信功能,提供了手机界面软件。通过手机界面软件可以控制CE装置的电泳运行,实时接收C^4^D发出的数据信息,显示电泳图谱并进行数据处理,检测消毒剂中2种季铵盐,线性关系良好、LOD低、重复性好、准确性高且便于携带,可用于消毒液中季铵盐现场定量检测。张丕旺等^[69]^采用多材料3D打印技术研制了用于毛细管电泳的电容耦合非接触电导检测-激光诱导荧光二合一检测池(C^4^D-LIF),在实验室内实现复杂结构的制作,降低了制作成本,且便于方法的改进和推广。罗芳等^[70]^建立了CZE和离子淌度经验公式测定洛伐他汀的绝对淌度*m*_0_和酸度系数p*K*_a_值的新方法,适用于酸性和碱性分析物*m*_0_和p*K*_a_等理化参数的测定。韩诗邈等^[71]^利用CE结合同步竞争法和多轮筛选法筛选得到了8-氧代鸟嘌呤DNA糖基化酶的核酸适配体。

### 4.2 《分析化学》

浙江大学与北京大学医学部^[72]^合作发表了“基于同轴鞘流的毛细管电泳-电喷雾质谱接口的研究与应用”,通过设计不同的电路连接方式,实现了自制接口与两台具有不同结构ESI源的质谱仪器的在线联用,研制了基于双毛细管的伸出型同轴鞘流毛细管电泳-电喷雾电离质谱接口,制作简单,死体积小(低至4 pL),易于操作(可在10 min内完成接口的组装和CE-MS系统的构建)。适用于超微量样品甚至单细胞样品的分离分析,体现了我国在CE-MS接口的研究现状,为实现CE-ESI-MS接口的商品化提供了有效途径。

## 5 结论

纵观全年,CE依旧在临床研究、细菌、病原体、医用蛋白质和药物分析方面发挥优势;CE-MS的新应用、硬件开发和联用仍是热点。以新材料为核心的各种增敏技术、以3D打印-CE-MS为代表的可视化技术,将是未来研究的“热点”。

以上内容难免有遗漏和不妥之处,请广大学界同仁及应用从业人员批评指正。
